# Improving Vaccination Coverage Through Community Pharmacy Service Delivery in Nigeria: The COVID‐19 Experience and Implications for Policy Review

**DOI:** 10.1002/hcs2.130

**Published:** 2025-02-17

**Authors:** Yejide Olukemi Oseni, Ukamaka Gladys Okafor, Taofik Oladipupo Odukoya, Hamidu Adediran Oluyedun, Abiodun Abdulah Ajibade, Yusuff Olanrewaju Azeez, Abigail Isaac Okonu, Oladapo Adewale Adetunji, Adebusuyi Akande Ademisoye, Fatimah Adebukola Sanusi, Okechi Eberechukwu Nzedibe

**Affiliations:** ^1^ Department of Clinical Pharmacy and Pharmacy Administration Faculty of Pharmacy Lead City University Ibadan Nigeria; ^2^ Department of Global Health and Bioethics EUCLID University Bangui Gambia; ^3^ Vanguard Pharmacy Ltd Ibadan Nigeria; ^4^ College of Health Sciences and Technology Ibadan Nigeria; ^5^ Alvid Pharmacy Ltd Ibadan Nigeria; ^6^ Faculty of Pharmacy University of Ibadan Ibadan Nigeria; ^7^ Pharmacy Council of Nigeria, Lagos Zonal Office Lagos Nigeria; ^8^ Department of Pharmaceutics Faculty of Pharmacy University of Ibadan Ibadan Nigeria; ^9^ Department of Pharmaceutical Chemistry, Faculty of Pharmacy Lead City University Ibadan Nigeria; ^10^ Department of Clinical Pharmacy and Biopharmacy Faculty of Pharmacy Olabisi Onabanjo University Ago Iwoye Ogun State Nigeria; ^11^ Histripes Pharmacy Uyo Nigeria

## Abstract

**Background:**

Globally, the use of community pharmacies and pharmacists in the delivery of vaccination services has been hampered by several factors, laws, and regulations that do not support pharmacists to participate in the delivery of vaccination services. With the advent of COVID‐19 pandemic, many countries have included community pharmacists and pharmacies in vaccination services to improve coverage. This study described the delivery of vaccination services in community pharmacies using the COVID‐19 experience and how their involvement impacted vaccination coverage in Nigeria. It also exposed how this experience can be used to support policy revisions to formally recognize pharmacists in immunization delivery.

**Methods:**

A descriptive cross‐sectional study was conducted among 474 community pharmacists in two southwestern States in Nigeria, using a semi‐structured questionnaire. It determines the number of community pharmacists who have been trained in the delivery of vaccination services, the types of vaccination services provided, and vaccines administered in their pharmacies. Data were analyzed with descriptive and inferential statistics and *p*‐value at ≤ 0.05.

**Results:**

Response rate was 86.7%. Less than half of the respondents (40.1%) had undergone vaccination training. Of the 129 (31.4%) respondents that provide vaccination services, 72 (55.8%) administer vaccines in their pharmacies. Out of these 72 respondents; 45 (62.5%) were administering vaccines before their involvement in COVID‐19 vaccine administration; 57 (79.2%) of the health personnel who administer vaccines were pharmacists; 60 (83.3%) of them administer vaccines on request; 22 (30.6%) administered COVID‐19 vaccines only; and only 7 (9.7%) of the respondents had administered over 500 doses of COVID‐19 vaccines. Training in vaccination was associated with the vaccination services provided (*p* < 0.05). Respondents suggested government support through legal framework and policy review, training and empowering pharmacists in vaccine administration, and recognition of community pharmacists as PHC providers.

**Conclusion:**

Training of community pharmacists in vaccination services had increased the number of respondents' involvement in the delivery of the services while the delivery of COVID‐19 vaccination by community pharmacists had increased the number of clients vaccinated, hence improved coverage in Nigeria. Also, policy review and inclusion of community pharmacists and pharmacies in the national database will improve vaccination coverage and immunization service delivery in Nigeria.

## Graphical abstract

This study described the delivery of vaccination services in community pharmacies among 474 community pharmacists. With a response rate of 86.7%, less than half of the respondents (40.1%) had undergone vaccination training, 129 (31.4%) provide vaccination services while 72 of 129 (55.8%) administer vaccines in their pharmacies. Government support through legal framework and policy review will improve pharmacists as vaccinators and coverage.

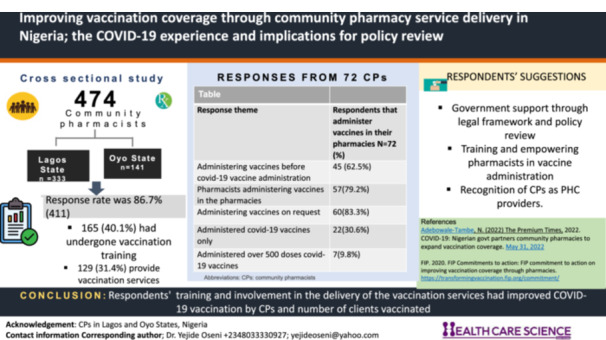

**Abbreviations**
FIP: International Pharmaceutical Federation
LGAs: local government areas
PCN: Pharmacy Council of Nigeria

## 1 Background

Vaccination coverage is an outcome immunization indicator commonly used in monitoring immunization programmes. It is used to measure the number of people who received a defined number of doses for a certain vaccine out of the total number of people for which the vaccine was intended [1]. Despite tremendous progress in the past few years, vaccination coverage has plateaued in recent years and dropped since 2020. Global coverage dropped from 86% in 2019 to 81% in 2021. The COVID‐19 pandemic and associated disruptions over the past few years have strained health systems, with 25 million children missing out on vaccination in 2021. This is higher by six million than what was obtainable in 2019 and the highest number since 2009 [2].

By early 2022 one billion doses of COVID‐19 vaccine had been delivered through COVID‐19 Vaccines Global Access Initiative (COVAX) [2]. Globally, as of February 28, 2023, a total of 13,228,728,467 vaccine doses have been administered [3]. While 69.7% of the world population has received at least one dose of a COVID‐19 vaccine, only 27.9% of people in low‐income countries have received at least one dose [4]. In Nigeria, WHO reported an estimated 6.2 million children that missed out on receiving routine vaccines between 2019 and 2022 due COVID‐19 pandemic; the government in collaboration with WHO and partners such UNICEF and GAVI are intensifying strategies to reduce unimmunized children by 15% and 80% by 2024 and 2028, respectively [5].

Pharmacists are highly trusted health professionals who practice in all healthcare settings throughout the world, and are highly accessible across all the communities [6]. The International Pharmaceutical Federation (FIP) stresses the critical role that pharmacy teams can play in immunizing patients and populations and contributing to improving vaccination coverage in multiple ways, including evidence‐based counseling and advocacy. FIP also stressed that accelerating vaccine access to pharmacy teams on the front lines contributes to widening access to vaccines for all. The provision of vaccination services through pharmacies widens access to vaccines for everyone, especially hard‐to‐reach population groups and those in medically underserved communities [7]. This is because pharmacies remain the most accessible health facilities despite the challenges brought about by the pandemic [8, 9].

The FIP also encouraged pharmacists and its member organization to expand the regulatory scope of practice of appropriately trained and certified pharmacists to authorize them to administer a broad range of vaccines beyond infancy [10]. Also to promote the competence of pharmacists in vaccine administration through the development of the required knowledge and skills as an integral part of pharmacists' foundational education and training, and/or through continuing professional development opportunities [10]. This is to accelerate vaccination equity, access, and sustainability [7].

The COVID‐19 pandemic experience has facilitated the inclusion of community pharmacists (CPs) as vaccinators in most countries of the world including the USA, Canada, Switzerland, Saudi Arabia, Jordan, and so forth [8, 10, 11, 12, 13, 14]. In Nigeria, CPs also through their professional organization were committed to driving their inclusion as primary health care (PHC) provider to expand their role in vaccination whereby some CPs were able to gain knowledge and skill in vaccine administration through different engagements in knowledge and skill acquisition from corporate industry‐led, FIP vaccination masterclass and through training organized in collaboration with Mercer University, USA [15]. The evidence‐based practice of CPs during COVID‐19 pandemic has been an avenue for pharmacists to present their case to the government for their inclusion as vaccination centers with the view to increase vaccination coverage in Nigeria [16]. This has driven a policy review in recent times. In a statement jointly signed by the Pharmaceutical Society of Nigeria (PSN) and the Association of Community Pharmacists of Nigeria (ACPN) in November 2021, the association hailed the Federal Government for including community pharmacies as COVID‐19 vaccination centers to reduce vaccine hesitancy [15]. Further to this, on May 31, 2022, the executive director of the National Primary Health Care Development Agency (NPHCDA), announced that the Nigerian government had co‐opted community pharmacies into COVID‐19 vaccination campaign to enable more access to vaccines among rural communities. This, he said will ensure vaccine availability to tackle the virus which has claimed millions of lives globally [17]. The roles of pharmacists in filling the low vaccine coverage gap were found to be favorable with influenza vaccines in the United States of America and some high‐income countries. Therefore, investigating the impact of CPs in other countries, especially developing ones due to their expanding roles globally, is germane [18].

Most of the studies conducted in Nigeria were based on perception and willingness to administer vaccination in community pharmacies. The involvement of CPs in COVID‐19 vaccine administration commenced in June 2022 after the approval by the federal government and no study had been conducted to assess the involvement of CPs in the delivery of immunization program. Hence, this study is novel. This study described the delivery of vaccination services in community pharmacies in Nigeria using the COVID‐19 experience. In addition, this survey assessed the improvement in vaccination coverage as a result of the involvement of community pharmacies and how this community service by pharmacists can be strengthen in policy review and legally position CPs in immunization program in Nigeria.

## 2 Research Methods

### 2.1 Research Design

This study is a descriptive cross‐sectional research focused on documenting the vaccination practices of CPs in Lagos and Oyo State, Nigeria and was conducted in 2023. It emphasizes external factors such as demographic characteristics, training in vaccination services, and the types of vaccines administered, without delving into the cognitive processes or motivations behind these practices. The research employs structured questionnaires and statistical methods to analyze observable and measurable data, providing empirical insights into vaccination service delivery among CPs. While the study effectively captures the extent of involvement and factors influencing vaccination practices, it does not explore the underlying intentions or cognitive processes driving these behaviors. Instead, it offers a detailed assessment of the current state of vaccination services and identifies gaps for potential improvement, serving as a foundation for further research into the “why” and “how” of pharmacists' engagement in vaccination services.

### 2.2 Study Area

Lagos and Oyo States are located in the southwest geo‐political zone of Nigeria. Oyo State has 33 local government areas (LGAs) and consists of both urban and rural communities. Ibadan which is the capital of Oyo State is the largest city in West Africa consists of 11 LGAs and out of the 33 LGAs in the State. It is the sixth populated State in Nigeria but second to Lagos State in population size in southwest, Nigeria [19]. Lagos is the economic and commercial capital of Nigeria. It consists of 20 LGAs. Though it is the smallest State in Nigeria by size, it has the second highest urban population in Nigeria [19]. The Lagos and Oyo States also house large populations of people living in underserved communities as well as urban‐rural communities. The largest concentration of CPs in Nigeria practice in Lagos State while Oyo State is third on the list in southwest, Nigeria, next to Ogun State [20].

CPs in Lagos and Oyo States have been previously involved in vaccination training through the pharmacy association and other partners like their counterparts from other States in Nigeria. However, the two States out of six states in southwest Nigeria were involved in vaccination services especially COVID‐19 vaccine administration after approval by the federal government and have received support from the partners in providing the services. Their practice and involvement during the COVID‐19 pandemic were added advantages in the consideration of the inclusion of community pharmacies and pharmacists in COVID‐19 vaccination services by the federal government.

### 2.3 Study Participants

The study participants were licensed CPs working in registered community pharmacies in Lagos and Oyo States, Nigeria. Registered community pharmacies with Pharmacy Council of Nigeria (PCN) and licenced CPs in Lagos and Oyo States were included in the study while non‐registered community pharmacies with PCN, CPs not licenced with the PCN, all locum, intern, and serving pharmacists were excluded.

### 2.4 Sample Size Determination

The PCN register as of December 31, 2022, showed that 2750 (44%) of registered community pharmacies and 3079 (38%) of CPs in Nigeria are located and practice in the southwest zone of Nigeria [20]. The PCN register also showed that 1986 and 217 community pharmacies were registered in Lagos and Oyo states respectively in the year 2022 while 2115 and 265 CPs were licensed in Lagos and Oyo States in the year 2022, respectively. Using the Taro Yamane formula to determine the sample size from the population of community pharmacies from each of the State, *n* = *N*/1 + *N*(*e*)^2^, where *n* = sample size, *N* = population size under study, *e* = margin of error put at 0.05; for Lagos State *N* = 1986, hence *n* = 1986/(1 + 1986 × 0.05^2^) = 333.

For Oyo State, *N* = 217, hence *n* = 217/(1 + 217 × 0.05^2^) = 141.

Hence, the total sample size for the two states = 333 + 141 = 474.

### 2.5 Sampling Method

**Figure 1:**
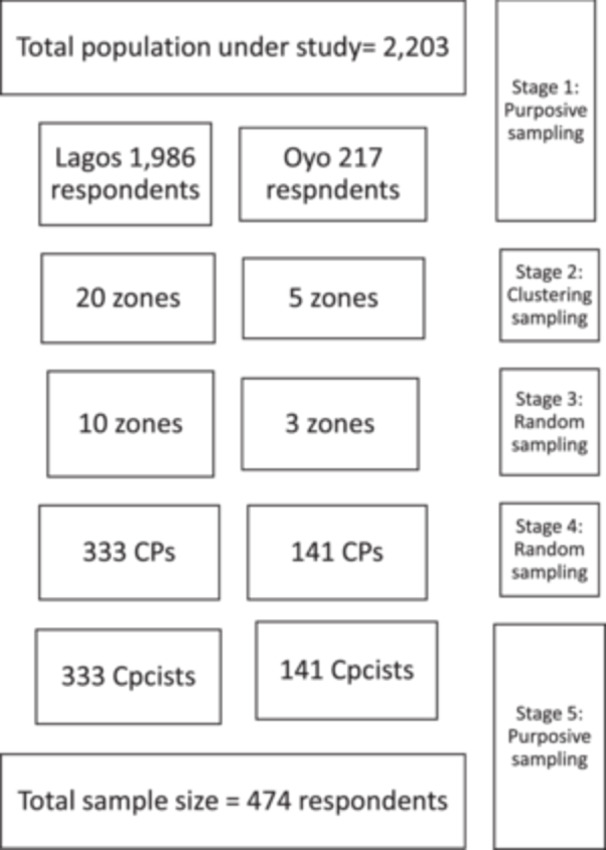
A multi‐stage sampling technique of community pharmacies and pharmacists. CPs, community pharmacies; Cpcist, community pharmacists.

A multi‐stage sampling technique was employed in this study as presented in Figure 1. The two States were purposefully selected because of the quality they possess on the issue of vaccination and COVID‐19 experiences. Community pharmacies in the States were clustered based on existing zonal arrangement of 20 and five zones for Lagos and Oyo state respectively. To have a good representation of each cluster especially in rural areas, random selection of the clusters was done in each state based on urban/rural distribution in line with each state definition. Ten zones were selected from Lagos and three zones from Oyo State. Community pharmacies were further randomly sampled from each selected zone/cluster. Lastly, a purposive sampling technique was used to select CPs from the selected community pharmacies based on the eligibility of the respondents. These include 333 and 141 CPs from Lagos and Oyo States respectively and only one community pharmacist per pharmacy was sampled.

### 2.6 Research Instruments

Semi‐structured, self‐administered questionnaire earlier piloted among CPs in another State in the southwest, Nigeria was used.

Questions were divided into three sections; section A surveyed the demographic characteristics of the respondents including age, gender, qualifications, years of post‐graduate pharmacy experience, and community pharmacy experience. This also includes the employment status, state of practice, and type of pharmacy among others. Section B sought to know if the respondents had been trained in vaccination services, the sources of training, if they provide vaccination services, and the type of services they provide. Section C questions were centered on the respondents who administer vaccines in their premises. It sought to know the health personnel that administer the vaccines, the types of vaccines administered, and the number of COVID‐19 vaccines administered as of the period of the survey. Respondents also made suggestions on how the involvement of community pharmacies in vaccination service delivery can be improved in Nigeria, using the COVID‐19 experience.

### 2.7 Reliability and Validity of the Research Instrument

The questionnaire was checked for content and construct validity. A group of researchers from different areas of pharmacy practice who are research experts appraised the questionnaire. A reliability test was conducted with the use of Cronbach's alpha value after the pretest of the questionnaire to measure the internal consistency.

### 2.8 Data Collection Procedure

Researchers and trained field assistants were involved in the administration of the questionnaire. The self‐administered questionnaire was done both with the use of Google Forms and hard copies for the CPs. Zonal meetings were attended to distribute the hard copies of the questionnaire. In like manner, researchers who were in some of the existing pharmacy association zonal groups distributed the Google Forms and follow up with reminders to the respondents. The responses were followed up through both WhatsApp and text messages as well as phone calls, and extra time frame was given to respondents in an attempt to receive a 100% response rate.

### 2.9 Data Analysis

The data generated were coded before analysis using Statistical Package for Social Sciences, SPSS (version 23) software. Descriptive statistics such as percentage, frequency, mean and standard deviation (SD) were used where applicable for categorical and continuous variables. Due to a number of possible independent variables, we first looked for associations between each categorical independent variables and the dependent variable (i.e., vaccination services provided) using cross tabulations and Chi‐squared tests to help decide which variables to include in logistic regression. The variables with association along with continuous independent variables were used to conduct logistic regression analysis to determine if any of them was a predictor of the vaccination services. *p*‐value was set at ≤ 0.05.

## 3 Results

Cronbach's alpha value of 0.791 obtained from the result of the pretest showed internal reliability of the survey instrument.

Out of the 474 respondents, 411 responses were received and analyzed, given a response rate of 86.7%. A total of 63 (13.3%) respondents could not deliver their questionnaire within the stipulated time frame despite all the efforts of the researchers. Out of the 411 responses analyzed, 296 (72.0%) respondents were received from Lagos State while 115 (28.0%) respondents were from Oyo State. The demographic characteristics of respondents are shown in Table 1. More than a quarter (27.7%) of the respondents were within 31–40 years of age, with a mean age of 43.06 ± 13.09, while 53.5% were male, 64.5% had B. Pharm./B.Sc. Pharm as the highest degree attained. Also, 52.6% were pharmacy owners, 87.1% were employed by independent pharmacies, and 42.6% had between 1 and 10 years' post‐graduation experience.

**Table 1:** Demographic characteristics of respondents.

Variable	*n* (%)	Mean ± SD
State of practice of respondents		
Lagos	296 (72.0)	
Oyo	115 (28.0)	
Age of respondents (years)		43.06 ± 13.09
21**–**30	90 (21.9)	
31**–**40	114 (27.7)	
41**–**50	88 (21.4)	
51**–**60	66 (16.1)	
> 60	53 (12.9)	
Sex		
Male	220 (53.5)	
Female	191 (46.5)	
Highest degree of education of respondents		
B.Pharm/B.Sc Pharm	265 (64.5)	
Pharm. D	23 (5.6)	
M.Sc./MBA/MPH/PGDs	90 (21.2)	
FPCPharm	27 (6.6)	
Ph.D	6 (1.5)	
No. of year postgraduation (years)		17.61 ± 12.58
1**–**10	175 (42.6)	
11**–**20	87 (21.2)	
21**–**30	72 (17.5)	
31**–**40	59 (14.4)	
41**–**50	17 (4.1)	
> 50	1 (0.2)	
No. of years of community pharmacy practice (years)		12.39 ± 10.20
1**–**10	235 (57.2)	
11**–**20	91 (22.1)	
21**–**30	52 (12.7)	
31**–**40	28 (6.8)	
> 40	10 (2.4)	
Employment status		
Employee pharmacists	195 (47.4)	
Pharmacy owners	216 (52.6)	
Type of pharmacy		
Independent	358 (87.1)	
Chain	53 (12.9)	

Abbreviation: SD, standard deviation.

The response theme on vaccination training and services provided by the respondents is presented in Table 2. Less than half (165, 40.1%) of the respondents had undergone vaccination training. The most common medium of training for the (165, 40.1%) respondents were professional association‐based training (64.8%), NGOs (26.1%), and webinars (23.6%). Only 31.4% (129) of the respondents provide vaccination services. The services provided by the 129 (31.4%) respondents ranged from education on the need to be vaccinated (101, 78.3%), referral to vaccination centers (75, 58.1%), administration of vaccines (72, 55.8%) and storage of vaccines (47, 36.4%).

**Table 2:** Response theme on vaccination training and services provided by respondents.

Response theme	*n* (%)
I have undergone vaccination training	165 (40.1)
Sources of training	***n*** = **165**
Webinar	39 (23.6)
Pharmacy profession association	107 (64.8)
Private provider	33 (20.0)
NGOs	43 (26.0)
LGAs	22 (13.3)
Others	15 (39.1)
I provide vaccination services	**129 (31.4)**
Type of vaccination services provided	***n*** = **129**
Administration of vaccine	72 (55.8)
Education on the need to be vaccinated	101 (78.3)
Referral to vaccination centers	75 (58.1)
Collaboration with other health center to vaccinate	45 (38.9)
Storage of vaccines	47 (36.4)

Abbreviations: LGAs, local government areas; NGOs, nongovernment organizations.

Presented in Table 3 is the description of vaccinator respondents. Out of the 72 vaccinator respondents, 45 (62.5%) were administering vaccines before their involvement in COVID‐19 vaccine administration. However, 57 (79.2%) of the health personnel who administer vaccines were pharmacists, 21 (29.2%) were registered trained nurses and 10 (13.9%) were community health workers (CHEW). Furthermore, 60 (83.3%) of them administered vaccines on request, 22 (30.6%) administered COVID‐19 vaccines only, and 7 (9.7%) had administered over 500 doses of COVID‐19 vaccine.

**Table 3:** Response from those administering vaccine in their pharmacies ONLY (*N* = 72).

Response theme (*N* = 72)	*n* (%)
I administered vaccine before covid‐19 pandemics in my pharmacy	45 (70.6)
Health personnel that administer the vaccines in my pharmacy	
Registered trained Nurse	21 (29.2)
Auxiliary Nurse	9 (12.5)
CHEW	10 (13.9)
Pharmacy technician	3 (4.2)
Pharmacists	57 (79.1)
Other healthcare professional in the pharmacy	7 (9.7)
Type of vaccines administered	
Routine immunization for children	6 (8.3)
COVID‐19 vaccine	22 (30.6)
All range of vaccines	14 (19.4)
Vaccines on request	60 (83.3)
Number of COVID‐19 vaccine doses administered	
1–100	6 (8.3)
101–500	5 (6.9)
501–1000	3 (4.2)
> 1000	4 (5.6)

Abbreviation: CHEW, community health workers.

Association between the categorical independent variables and the dependent variable (i.e. vaccination services provided) is as presented in Table 4. There are association between level of education, employment status, respondents who had undergone vaccination training and vaccination services provided.

**Table 4:** Cross tab of socio‐demographics characteristics with vaccination services provided.

Socio‐demographic characteristics	*df*	Vaccination services provided *n* = 129 *p*‐value
Sex	1	0.678^ns^
Level of education	4	0.032*
Employment status	1	0.050*
Type of community practice	1	0.166^ns^
State of practice	1	0.245^ns^
Undergone vaccination training	1	**< 0.001** *

Abbreviations: df, degrees of freedom; ns, not significant.

* Significant at **<0.001** *p*‐value. ^ns^is not significant.

In Table 5, the influence of respondents' characteristics on the provision of vaccination services by CPs is highlighted. Vaccination training (*β* = 2.120, 95% CI: 5.043–13.770) had the likelihood of positively influencing respondent's provision of vaccination services (*p* < 0.01). There is 80% likelihood of the provision of immunization services by CPs who had undergone vaccination training than those without training. However, the likelihood of vaccination services provision being influenced by employment status, highest level of education, years of postgraduation experience, years of community pharmacy practice, and age was insignificant (*p* > 0.05).

**Table 5:** Predictors of vaccination services provided by community pharmacists.

Variable				95% CI	
*N* = 411	Coefficient	*p*‐value	Exp (B)	Lower	Upper
Employment status					
Employee pharmacists	Reference category				
Pharmacist owners	0.325	0.270^ns^	1.384	0.777	2.464
Undergone vaccination training					
No	Reference category				
Yes	2.120	**< 0.001** *	8.334	5.043	13.770
Level of education					
Ph.D	Reference category 0.092				
B.Pharm	0.908	0.465^ns^	2.478	0.217	28.351
Pharm. D	0.034	0.980^ns^	1.035	0.070	15.218
Master's degrees/PGDs	0.427	0.734^ns^	1.533	0.130	18.042
Fellow WAPCP	1.649	0.208^ns^	5.200	0.400	67.677
Age (in years)	0.040	0.225^ns^	1.041	0.976	1.111
Year of experience postgraduation	**−**0.069	0.054^ns^	0.933	0.869	1.001
Year of experience in community pharmacy practice	0.011	0.645^ns^	1.011	0.964	1.060
Constant	**−**0.3467	0.021	0.031		

Abbreviations: CI, confidence interval; ns, not significant.

* Significant at **< 0.001** *p*‐value.

For improved vaccination coverage and administration through community pharmacies, 24.6% respondents crave for government support through legal framework and policy review, 11.6% of the respondents opined for inclusion of CPs in the national database, however, 5.4% requested proper recognition of pharmacists as PHC provider. Furthermore, 18% of the participants suggested training and empowering pharmacists in vaccine administration while 14.4% suggested provision of adequate facilities for pharmacists in vaccination projects as depicted in Table 6.

**Table 6:** Suggestions to government on the community pharmacy inclusion in vaccination in Nigeria.

Response theme	*N* (%)
Recognition of pharmacists as PHC providers	22 (5.4)
Encourage inclusion of pharmacists	49 (11.9)
Provision of adequate facilities for pharmacists in vaccination projects	59 (14.4)
Training and empowering pharmacists in vaccine administration	74 (18.0)
Government support through legal framework and policy	101 (24.6)
No comment	106 (25.8)

Abbreviation: PHC, primary health care.

## 4 Discussion

Vaccine have been instrumental in thwarting the spread of infectious diseases and have been a major global public health success [21]. Though, not many research works had been carried out in Nigeria regarding involvement of CPs in vaccination services, this study described the proportion of CPs that engage in vaccination services. It further described the proportion of CPs who have obtained training in the delivery of vaccination services, the types of vaccination services provided in their premises, the types of vaccines administered and their involvement in the delivery of COVID‐19 vaccines after the approval to community pharmacies in Nigeria. This study is part of the evidence‐based studies conducted to persuade the policymakers and regulators on the need to include CPs in the national database of immunization services and administration in Nigeria.

Training is a significant factor that influenced the engagement and involvement of CPs in vaccination services as obtained in this study. In a systematic review conducted in low‐and‐medium‐income countries [LMICs]), CPs were seen to be receiving training related to vaccination services [22]. In this study, about 40% of the respondents had undergone vaccination training. This is similar to the study conducted in Jordan where 50% of the community pharmacist respondents had received training in vaccination services [23] which was an indication of the pharmacists' readiness to deliver vaccination services.

This study showed that the professional association mostly spearheaded the organized training of her members in vaccination services and administration in Nigeria as earlier reported [15]. This is in compliance with previous study [24], which careful considered the need for effective partnership between public and private sectors in primary healthcare through community pharmacies, to increase the provision of immunization as part of the Expanded Programme on Immunization/National Programme on Immunization (EPI/NPI). The study further revealed that trainings were received through the NGO partners and Webinar participation. This is in line with previous study by Percy et al. [25] where CPs were trained in vaccination services through Webinars and onsite and through the train‐the‐trainer model to improve vaccination rate.

The provision of vaccination services by 31.4% of the pharmacies is an improvement to healthcare services and is similar to findings by a similar study by Alnahar et al. [23] where 36% of the CPs reported vaccinating patients in their pharmacies.

CPs in this study were found to provide range of services from education on the need to be vaccinated, referral to vaccination centers, administration of vaccines and storage of vaccines. In an assessment study on the extent of participation of CPs in immunization services in Cross River State, Nigeria before the COVID‐19 pandemic, respondents were willing to provide mainly education on immunization to the clients, administering vaccines to clients, and engaging in mass campaign on immunization [26]. Previous studies also showed that CPs had demonstrated the ability to administer vaccines as immunizers, as well as advocators, educators, and facilitators for vaccination administration [18, 22].

This study showed that participation in vaccination training was statistically significantly related with the provision of vaccination services by the respondents and was the only predictor to the provision of vaccination services. This is related to previous study where training has been shown to increase CPs' involvement in the delivery of vaccines and community pharmacy‐based vaccination services have increased both in the number of immunization providers and the number of sites where patients can receive immunizations [27].

The provision of vaccination services is geared to increase vaccination coverage and reduce vaccine hesitancy in the community they serve. This study showed that CPs were able to administer all ranges of vaccine even before COVID‐19 vaccine. CPs have been considered well‐trained health professionals while community pharmacies can serve as accessible locations for patient‐centered medication management services to enhance the health and wellness of the community [8]. The contributions pharmacists played in vaccination production, research and development, safety, and pharmacovigilance of vaccination is an indication for an increasing need for vaccination coverage through CPs in Nigeria [10, 28].

This study revealed that the involvement of the respondents in administration of vaccines also increased after the government's regulatory body approval of community pharmacies as COVID‐19 vaccination centers and most of the vaccinators were pharmacists [17]. This was due to the consistent advocacy to the government by the professional body [15]. Studies have shown that vaccination services are feasible in community pharmacies [29, 30, 31] and CPs have assumed new roles in COVID‐19 testing and vaccination [13], while regulatory and professional bodies are all seen as influencers in supporting pharmacists as vaccinators [23]. Clients vaccinated in the community pharmacies might not have been vaccinated at all or they would have opted for other vaccination providers but due to easy access, opening hours, and perceived trust [14, 32].

The introduction of a policy for pharmacist administration of influenza vaccine was seen to be associated with a modest increase in coverage in Canada [33]. The inclusion of CPs and pharmacies into COVID‐19 vaccination was approved in June 2022 when the delivery was already low and many did not see the reason to take the vaccine again. Regardless, in an interview with the principal technical advisor for the USAID Medicines, Technologies, and Pharmaceutical Services (MTaPS) COVID‐19 program in Nigeria, Enejoh [34] reported in August 2023 that more than 70,000 COVID‐19 vaccines were delivered by community pharmacies. This result is similar to the study in Switzerland where CPs had been involved in the delivery from the onset of the pandemic and were able to administer 68,169 COVID‐19 vaccines by June 2021 [14]. The engagement of the partners with the government regulatory bodies and the pharmacy association also helped in the improvement of the coverage where one of the States under study (i.e., Oyo State) was seen to have administered the highest number of COVID‐19 vaccines [32].

CPs' inclusion in vaccination and immunization services had long been overdue in Nigeria [24]. In a study by Yemeke et al. among LMICs in 2021, the legal administration of vaccines by CPs was reported in a few countries, Nigeria not inclusive [22]. The evidence‐based practice of CPs during COVID‐19 pandemic [16], their readiness as vaccinators which is evidenced in their participation in training even before the emergence of COVID‐19 pandemic, and the delivery of vaccinations before and after approval as seen from this study corroborated this. Also, the need for herd immunity and improvement in vaccination coverage of COVID‐19 vaccination has called for the inclusion of CPs in Nigeria like in other countries of the world in vaccine delivery [17, 34].

Previous studies in Nigeria had recommended the inclusion of CPs as PHC providers as they were able to deliver health promotion and point‐of‐care services while few were also involved in vaccination services [13, 35, 36, 37, 38]. The Pharmacy Professional Association has been at the forefront of bringing this to pass and the inclusion into COVID‐19 vaccination only by the federal government is just a stepping stone to bring this into reality. Hence, respondents suggested the need for a policy review for the inclusion of CPs in routine immunization in Nigeria and not just COVID‐19 vaccination. They also suggested the need for training and empowerment of CPs with adequate facilities for delivery. This is also related to the study conducted in Jordan where influencing factors to delivery of vaccination services by CPs included regulatory and Ministry of Health supports as well as provision of equipment/tools for delivery [23].

The study was conducted in two States out of 36 States in Nigeria, which may limit its applicability to other States of Nigeria with differing healthcare infrastructure, regulatory environments, or vaccination coverage. Also, the findings may not be generalizable to community pharmacies in states with fewer resources or different population demographics.

Though pretested, the semi‐structured questionnaire might not have captured all relevant variables affecting vaccination services, limiting the depth of data collected.

The multi‐stage and purposive sampling methods, while structured, could introduce biases in the selection of respondents. CPs more actively involved in vaccination services may have been disproportionately represented.

The 63 non‐respondents represent a small but notable proportion (13.3%) of the target sample and this is unlikely to bias the findings of this study significantly. Although there is no agreed standard for acceptable minimum response rate, a response rate of 75% and above is generally accepted for a descriptive study [39]. Therefore, we proceeded with the analysis for the 411 (86.7%) responses received within the stipulated time frame.

## 5 Conclusion

This study revealed that the training of CPs in vaccination services had contributed to the delivery of vaccination services, vaccine access and uptake in Nigeria. Within the short period of approval of community pharmacies delivery in COVID‐19 vaccination, CPs showed readiness to deliver vaccines in their pharmacies and there was increased involvement of community pharmacies in vaccination services and in administration of COVID‐19 vaccines, which led to improved vaccination coverage in Nigeria. The outcome of this study is an evidence‐based practice that vaccination services are feasible in community pharmacies through CPs. Therefore, the need to review policies for the inclusion of CPs and pharmacies in the national database of vaccinators.

Further study will qualitatively explore the strategies used by the pharmacy association in the inclusion of their members in the national database for all vaccination services in Nigeria using the COVID‐19 experience, and the challenges encountered in the process through focus group discussion and in‐depth interviews while the viewpoints of the partners, policymakers and government regulatory bodies to this inclusion for future consideration will be done through key informant interviews. Furthermore, suggested further study will evaluate h**o**w community pharmacy involvement in vaccination has changed since the COVID‐19 vaccine was authorized in Nigerian community pharmacies compared to before the COVID‐19 vaccine was authorized in Nigerian community pharmacies.

## Author Contribution

**Yejide Olukemi Oseni:** conceptualization (lead), data curation (lead), formal analysis (lead), funding acquisition (equal), investigation (lead), methodology (lead), project administration (lead), resources (equal), software (equal), supervision (lead), validation (lead), visualization (equal), writing–original draft (lead), writing–review and editing (lead). **Ukamaka Gladys Okafor:** conceptualization (equal), data curation (equal), formal analysis (equal), funding acquisition (equal), investigation (equal), methodology (equal), project administration (equal), resources (equal), software (supporting), supervision (equal), validation (equal), visualization (equal), writing–original draft (equal)writing–review and editing (equal). **Taofik Oladipupo Odukoya:** conceptualization (equal), data curation (equal), formal analysis (supporting), funding acquisition (equal), investigation (equal), methodology (supporting), project administration (equal), resources (equal), software (supporting), supervision (supporting), validation (supporting), visualization (equal), writing–original draft (supporting), writing–review and editing (supporting). **Hamidu Adediran Oluyedun:** conceptualization (equal), data curation (equal), formal analysis (supporting), funding acquisition (equal), investigation (supporting), methodology (equal), project administration (equal), resources (equal), software (supporting), supervision (supporting), validation (equal), visualization (lead), writing–original draft (supporting), writing–review and editing (supporting). **Abiodun Abdulah Ajibade:** conceptualization (supporting), data curation (equal), formal analysis (equal), funding acquisition (equal), investigation (equal), methodology (supporting), project administration (equal), resources (equal), software (equal), supervision (supporting), validation (supporting), visualization (supporting), writing–original draft (supporting), writing–review and editing (supporting). **Yusuff Olanrewaju Azeez:** conceptualization (equal), data curation (equal), formal analysis (equal), funding acquisition (equal), investigation (supporting), methodology (supporting), project administration (equal), resources (equal), software (equal), supervision (equal), validation (equal), visualization (equal), writing–original draft (supporting), writing–review and editing (supporting). **Abigail Isaac Okonu:** conceptualization (supporting), data curation (equal), formal analysis (supporting), funding acquisition (equal), investigation (supporting), methodology (supporting), project administration (equal), resources (equal), software (supporting), supervision (equal), validation (equal), visualization (supporting), writing–original draft (supporting), writing–review and editing (supporting). **Oladapo Adewale Adetunji:** conceptualization (supporting), data curation (supporting), formal analysis (supporting), funding acquisition (equal), investigation (supporting), methodology (supporting), project administration (equal), resources (equal), software (supporting), supervision (supporting), validation (supporting), visualization (supporting), writing–original draft (supporting), writing–review and editing (supporting). **Adebusuyi Akande Ademisoye:** conceptualization (supporting), data curation (supporting), formal analysis (supporting), funding acquisition (supporting), investigation (supporting) methodology (supporting), project administration (supporting), resources (supporting), software (supporting), supervision (supporting), validation (supporting), visualization (supporting), writing–original draft (supporting), writing–review and editing (supporting). **Fatimah Adebukola Sanusi:** conceptualization (supporting), data curation (supporting), formal analysis (supporting), funding acquisition (supporting), investigation (supporting), methodology (supporting), project administration (supporting), resources (supporting), software (supporting), supervision (supporting), validation (supporting), visualization (supporting), writing–original draft (supporting), writing–review and editing (supporting). **Okechi Eberechukwu Nzedibe:** conceptualization (supporting), data curation (supporting), formal analysis (supporting), funding acquisition (supporting), investigation (supporting), methodology (supporting), project administration (supporting), resources (supporting), software (supporting), supervision (supporting), validation (supporting), visualization (supporting), writing–original draft (supporting), writing–review and editing (supporting).

## Ethics Statement

The Oyo State Research Ethical Review Committee, Ministry of Health, gave ethical clearance (Reference no. AD 13/479/301) for the study.

## Consent

All participants provided written informed consent at the time of entering this study.

## Conflicts of Interest

The authors declare no conflicts of interest.

## Data Availability

The data that support the findings of this study are available on request from the corresponding author. The data are not publicly available due to privacy or ethical restrictions.
